# The YPLGVG sequence of the Nipah virus matrix protein is required for budding

**DOI:** 10.1186/1743-422X-5-137

**Published:** 2008-11-10

**Authors:** Jared R Patch, Ziying Han, Sarah E McCarthy, Lianying Yan, Lin-Fa Wang, Ronald N Harty, Christopher C Broder

**Affiliations:** 1 Department of Microbiology and Immunology, Uniformed Services University, Bethesda, Maryland 20814, USA; 2 Department of Pathobiology, School of Veterinary Medicine, University of Pennsylvania, 3800 Spruce St, Philadelphia, PA 19104-6049, USA; 3 CSIRO Livestock Industries, Australian Animal Health Laboratory, Geelong, Victoria 3220, Australia; 4 Plum Island Animal Disease Center, Agricultural Research Service, USDA, Greenport, NY 11944, USA; 5 Fox Chase Cancer Center, 333 Cottman Avenue, Philadelphia, PA 19111-2497, USA; 6 United States Army Research Institute of Infectious Diseases, Virology Division, 1425 Porter Street, Fort Detrick, MD 21702, USA

## Abstract

**Background:**

*Nipah virus *(NiV) is a recently emerged paramyxovirus capable of causing fatal disease in a broad range of mammalian hosts, including humans. Together with *Hendra virus *(HeV), they comprise the genus *Henipavirus *in the family *Paramyxoviridae*. Recombinant expression systems have played a crucial role in studying the cell biology of these Biosafety Level-4 restricted viruses. *Henipavirus *assembly and budding occurs at the plasma membrane, although the details of this process remain poorly understood. Multivesicular body (MVB) proteins have been found to play a role in the budding of several enveloped viruses, including some paramyxoviruses, and the recruitment of MVB proteins by viral proteins possessing late budding domains (L-domains) has become an important concept in the viral budding process. Previously we developed a system for producing NiV virus-like particles (VLPs) and demonstrated that the matrix (M) protein possessed an intrinsic budding ability and played a major role in assembly. Here, we have used this system to further explore the budding process by analyzing elements within the M protein that are critical for particle release.

**Results:**

Using rationally targeted site-directed mutagenesis we show that a NiV M sequence YPLGVG is required for M budding and that mutation or deletion of the sequence abrogates budding ability. Replacement of the native and overlapping Ebola VP40 L-domains with the NiV sequence failed to rescue VP40 budding; however, it did induce the cellular morphology of extensive filamentous projection consistent with wild-type VP40-expressing cells. Cells expressing wild-type NiV M also displayed this morphology, which was dependent on the YPLGVG sequence, and deletion of the sequence also resulted in nuclear localization of M. Dominant-negative VPS4 proteins had no effect on NiV M budding, suggesting that unlike other viruses such as Ebola, NiV M accomplishes budding independent of MVB cellular proteins.

**Conclusion:**

These data indicate that the YPLGVG motif within the NiV M protein plays an important role in M budding; however, involvement of any specific components of the cellular MVB sorting pathway in henipavirus budding remains to be demonstrated. Further investigation of henipavirus assembly and budding may yet reveal a novel mechanism(s) of viral assembly and release that could be applicable to other enveloped viruses or have therapeutic implications.

## Background

*Nipah virus *(NiV) and *Hendra virus *(HeV) are emerging members of the family *Paramyxoviridae *that are distinguished by their ability to cause fatal disease in both animal and human hosts, and comprise the genus *Henipavirus *[1,2]. HeV was recognized as a novel paramyxovirus in 1994 during an outbreak in eastern Australia that resulted in the death of one human as a consequence of virus transmission from infected horses. Another person later died from relapsed encephalitis as a result of HeV infection that was identified retrospectively [3]. Repeated HeV spillover events have since occurred five times, all involving horses, with the most recent occurrence in July 2008 which also involved two human cases, one of which was fatal [4,5]. NiV was identified during an outbreak of severe encephalitis in Malaysia and Singapore that began in 1998 and continued into 1999. In contrast to the HeV outbreak, this NiV episode involved hundreds of people and more than 100 deaths, with pigs serving as the intermediate amplifying host [6,7]. Since 1998 there have been 9 recognized occurrences of NiV infection of people, primarily in Bangladesh and India with the most recent in March 2008 [8-14]. The mortality in humans has been higher (~75%) in these spillover events, along with evidence of human-to-human transmission and the apparent lack of an intermediate host [8,15-17].

Several species of fruit bats (flying foxes) of the *Pteropus *genus serve as the primary natural reservoirs of HeV and NiV, although to date evidence of henipavirus infection in 5 other bat species across 5 genera has been reported (reviewed in [5]). NiV has been isolated from bat urine and partially eaten fruit, which suggests that it is relatively easy to obtain from the environment [18,19]. Indeed, direct transmission of NiV from flying foxes to humans from contaminated food sources has been suggested [9,20]. The Centers for Disease Control and Prevention (CDC) and the National Institute of Allergy and Infectious Diseases (NIAID) have classified HeV and NiV as priority pathogens, and work with live virus requires Biosafety Level-4 (BSL-4) containment.

Paramyxoviruses are enveloped viruses that replicate in the cytoplasm and contain a genome consisting of single-stranded negative-sense RNA [21]. The genome contains 6 principle genes: nucleocapsid (N), phosphoprotein (P), matrix (M), the fusion (F) and attachment (HN, H, or G) proteins, and the polymerase (L), along with accessory proteins that vary according to viral species [21]. The requirement for high containment conditions for working with live HeV or NiV has necessitated the development of recombinant protein expression systems as tools for elucidating details of the henipavirus life cycle. We previously established a virus-like particle (VLP) system in order to study the assembly and budding process of NiV, and determined that the M protein plays a central role in NiV assembly [22]. We also observed that expression of M alone resulted in the release of VLPs, as was also reported by Ciancanelli and Basler [22,23]. Other paramyxovirus M proteins with this property include Sendai virus (SeV) [24,25], human parainfluenza virus type 1 (hPIV-1) [26], and Newcastle disease virus (NDV) [27]. In contrast, simian virus 5 (SV5) (parainfluenza virus 5 (PIV5)) [28] requires expression of M along with N and either F or HN to produce VLPs [29]. The mechanism(s) that govern the budding of M remain unknown. A current area of interest in enveloped virus assembly and morphogenesis is the contribution of L-domains, which are protein motifs first identified in retroviral Gag precursor molecules that are important for late steps in assembly and budding (reviewed in [30-32]). L-domains interact with components of cellular machinery involved in multivesicular body (MVB) formation and are thought to commandeer those proteins for use in viral budding. The involvement of L-domains in virus assembly and budding has been extended to other enveloped virus families including arenaviruses [33], filoviruses [34,35], rhabdoviruses [35-37], and paramyxoviruses [38,39]. In certain cases, different L-domains can be functionally interchanged or mediate their activity in a position-independent manner within the protein molecule; however, these properties are not universal and it is now apparent that the surrounding regions or context within which the L-domain motif lies can be important for its function [31,40-42]. There are several well-characterized examples where the mutation or removal of a viral L-domain motif within, for example, the M protein, will abrogate the protein's ability to bud from expressing cells [35,38,43-45].

L-domain amino acid motifs that have been identified (along with the MVB protein each interacts with) include: P(T/S)AP (Tsg101), PPxY (Nedd4-like E3 ubiquitin ligases), YP(x)_n_L (AIP1/Alix), and ØPxV (none identified), where x is any amino acid and Ø is any aromatic amino acid [30-32]. Until the identification of the novel FPIV sequence in SV5 M [38], paramyxoviruses were not known to utilize L-domains in their assembly and morphogenesis. However, the M protein of many paramyxoviruses, including NiV and HeV, do not contain any identified L-domains, including the SV5 FPIV motif [38]. The MVB protein AIP1/Alix was shown to help facilitate SeV virion and VLP release through interactions with an undetermined sequence in the C protein, as well as through a recently identified YLDL sequence in the M protein [39,46]; however, a conflicting study failed to find a role for AIP1/Alix in SeV virion production [47]. Ciancanelli and Basler reported that NiV M contains a sequence, YMYL, that is required for VLP budding and, based on its ability to complement Ebola VP40 VLP formation, suggested that this sequence serves as an L-domain [23].

In this study, we report the identification of an amino acid sequence motif (YPLGVG) that is required for NiV and HeV M budding, and that appeared to partially complement the native Ebola VP40 phenotype but was VPS4-independent. However, complementation of the Ebola VP40 mutant was observed only in its effects on cellular morphology, characterized by extensive filamentous projections, and not in Ebola VP40 budding. In addition, cells expressing wild-type NiV M were noted to have a cellular morphology similar to that of VP40 expressing cells, and deletion of the YPLGVG sequence resulted in abrogation of this morphology and nuclear localization of M.

## Results

### Mutation of the YPLGVG motif in NiV matrix abrogates budding

**Figure 1:**
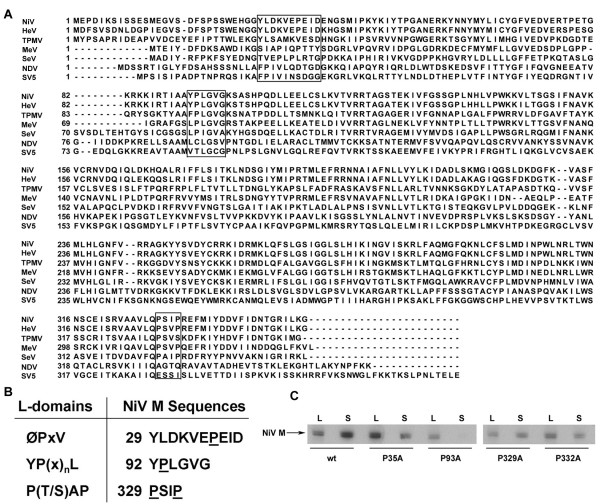
**Site-directed proline mutagenesis**. (A) ClustalW alignment of the M proteins from Nipah virus (NiV), Hendra virus (HeV), Tupaia paramyxovirus (TPMV), measles virus (MeV), Sendai virus (SeV), Newcastle disease virus (NDV), and simian virus 5 (SV5). Sequences of interest are boxed. Alignment was performed as described in the Methods. (B) Known L-domains are shown along with corresponding hypothetical L-domain sequences of NiV M (boxed in A). Underlined proline residues were mutated to alanine. (C) Mutant NiV M proteins, along with wild-type, were expressed in cells and released protein was pelleted through 10% sucrose. Proteins derived from the cell lysate (L) or culture supernatant (S) were immunoprecipitated using MAb F45G5 and analyzed by SDS-PAGE followed by autoradiography as described in the Methods.

Most L-domains described to date contain one or more proline residues. Because the NiV M protein does not contain any of the exact known L-domain motifs, we examined the entire M protein sequence for proline residues with surrounding amino acids that we considered to be similar to known L-domains. Following this analysis, we identified 3 sequences of interest with the intent of mutating the proline residues within these putative motifs to alanine (Fig. 1A and 1B). Residue P35 was targeted because it aligned closely to the SV5 FPIV (ØPxV) motif, although there were no further sequence similarities. P93 is located in a sequence similar to the YP(x)_n_L motif, and the P329 and P332 residues were targeted because of sequence similarity to the P(T/S)AP motif. It was also noted that P93, P329 and P332 are all well conserved among paramyxovirus M proteins but are absent in SV5 M (Fig. 1A), which further suggested that these proline residues might be part of an L-domain.

**Figure 2:**
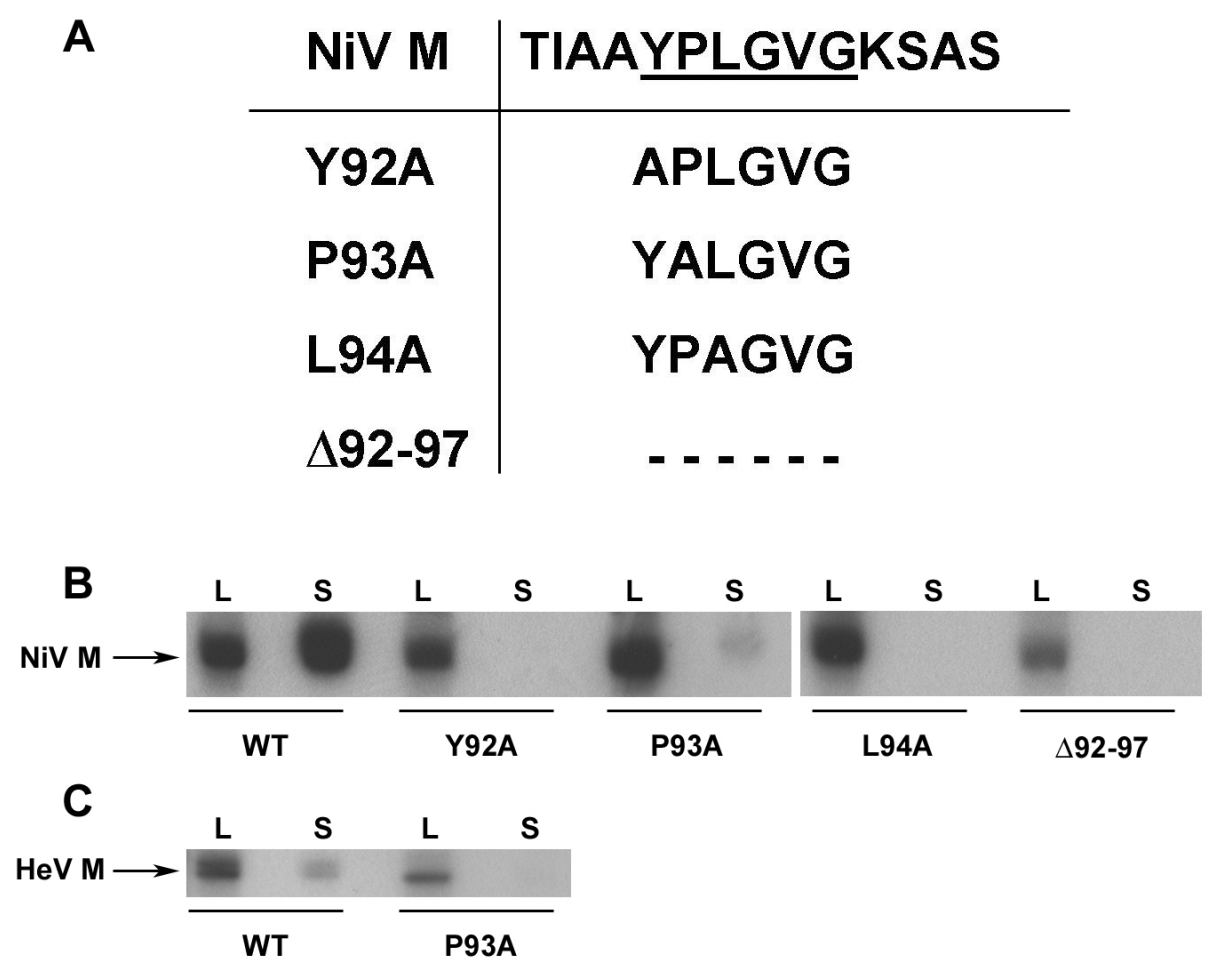
**NiV M sequence required for budding and possible late domain element**. (A) NiV M mutants were constructed with single alanine substitution mutations in the YPLGVG sequence (underlined), or with the whole sequence deleted (dashes). (B) Wild-type NiV M and the mutants depicted in A were all expressed in cells and released protein was pelleted through a 20% sucrose cushion. Proteins derived from the cell lysate (L) or culture supernatant (S) were immunoprecipitated with MAb F45G5 and analyzed by SDS-PAGE followed by autoradiography. (C) Wild-type and mutant P93A HeV M were expressed in cells and a budding assay was performed as described in the Methods.

The mutant M gene cassettes were expressed in cells, pelleted through a 10% sucrose cushion, and analyzed by immunoprecipitation and SDS-PAGE followed by autoradiography. The result of this comparison revealed that each of the mutant M proteins retained budding ability except for the P93A mutant, which exhibited a marked reduction in M protein release (Fig. 1C). The P93 residue is part of an amino acid sequence (YPLGVG) that resembles the YP(x)_n_L motif and also contains residues that are well conserved among paramyxovirus M proteins (Fig. 1A). To confirm this observation and further characterize the role of this sequence in M protein release, additional M mutants were constructed: Y92A, L94A, and Δ92–97 (Fig. 2A), that were then tested in the budding assay. The result of this experiment revealed that none of the additional mutant M proteins, with the exception of the P93A mutant, which sometimes retained a low level of budding activity, were released from expressing cells (Fig. 2B). All of the mutants were expressed at levels similar to wild-type NiV M, and these findings confirm the importance of this motif. We previously observed that HeV M is also released into the culture supernatant when expressed alone, although less efficiently than NiV M (unpublished observation). To determine whether the conserved YPLGVG sequence is required for HeV M release, we constructed HeV M P93A and Δ92–97 mutants and tested them in the budding assay. In contrast to wild-type HeV M, which was released into the supernatant, we did not detect release of the P93A mutant (Fig. 2C). We were unable to detect expression of HeV M Δ92–97 (data not shown), possibly due to mis-folding and degradation. These data suggest that the YPLGVG sequence is required for NiV and HeV M budding, and may also play a role in HeV M stability.

### The YPLGVG matrix motif can partially restore a defective VP40 phenotype

**Figure 3:**
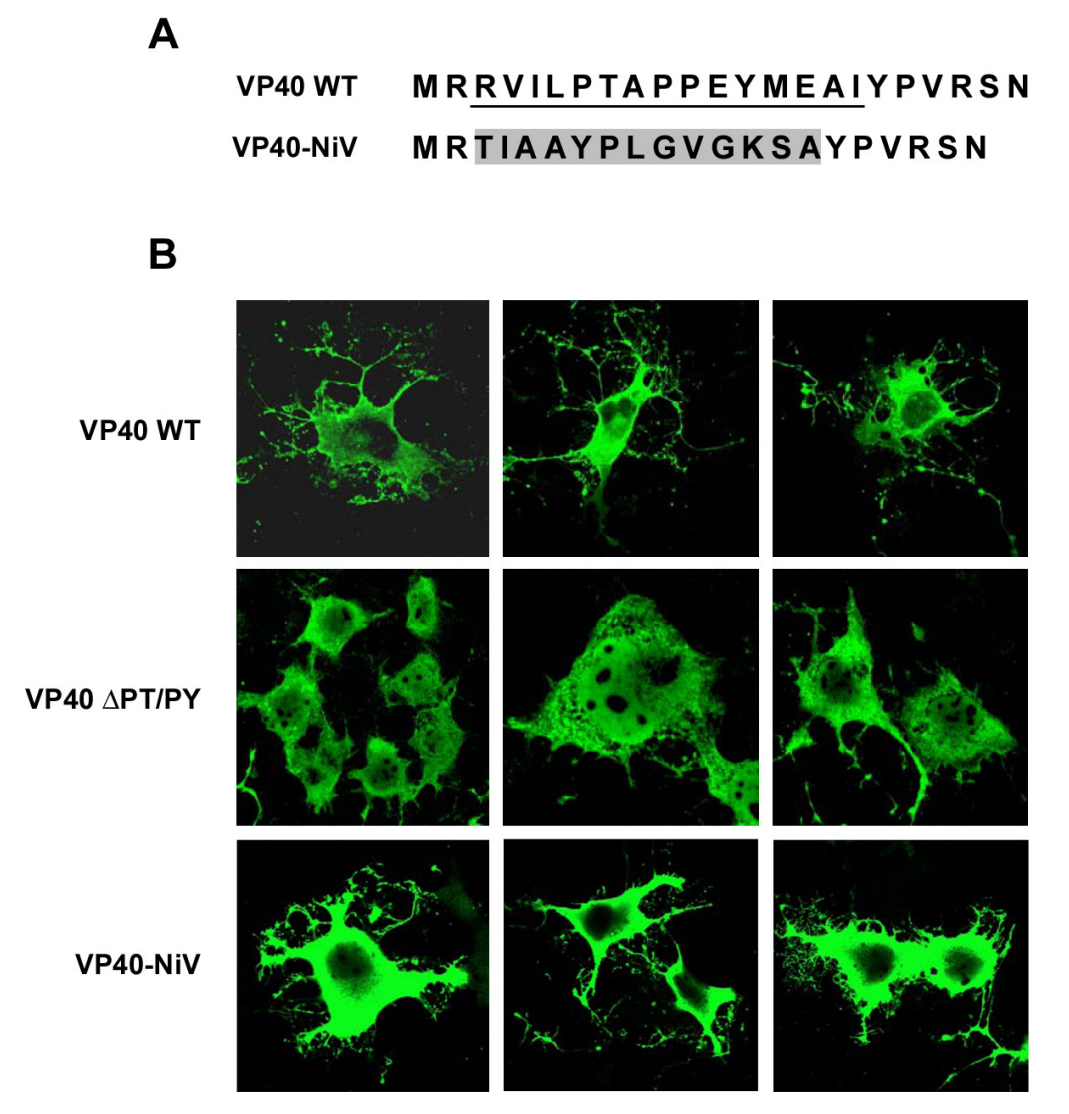
**NiV sequence can rescue Ebola VP40-induced cellular morphology**. (A) The L-domain and flanking sequence of VP40 (underlined) was replaced with a sequence derived from NiV M (shaded) containing the YPLGVG sequence. (B) COS-1 cells were transfected with plasmids encoding VP40 wt, VP40 ΔPT/PY, or VP40-NiV. Cells were fixed 20–24 h post-transfection, permeablilized, and incubated with mouse anti-VP40 MAb followed by Alexa Fluor 488 donkey anti-mouse antibody and analyzed by confocal microscopy. The VP40-NiV immunofluorescence experiment was performed separately; all images are representative.

**Figure 4:**
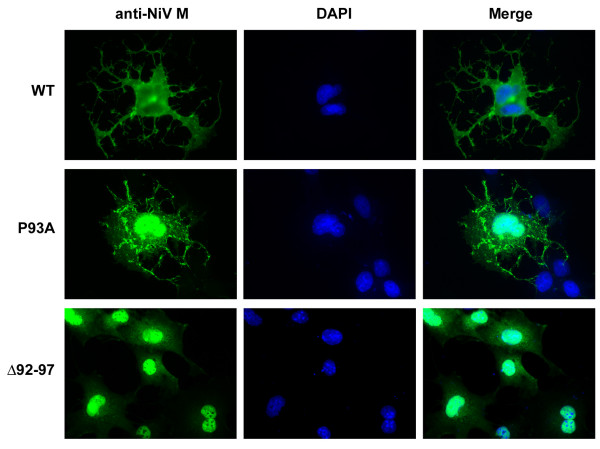
**Cellular morphology of NiV M-expressing cells is dependent on YPLGVG**. COS-1 cells were transfected with WT, P93A, or Δ92–97 NiV M plasmids and treated as described in Fig. 3B and the Methods except MAb F45G5 (anti-NiV M) was used, and cells were visualized by fluorescent microscopy.

A notable feature of most viral L-domain motifs is their inherent transferability to other M proteins defective in budding [31,38,44]. Therefore, we sought to determine whether this newly identified NiV M sequence could impart budding activity within the context of a different viral background. The Ebola VP40 protein contains two overlapping L-domain motifs, the PTAPPEY sequence, and deletion of these L-domains renders the protein defective in release [35]. A VP40 mutant was constructed in which we replaced the native L-domains and flanking residues with the NiV M protein YPLGVG motif (Fig. 3A), and this mutant was tested for budding activity. The results of this experiment revealed that, although some budding was evident, the NiV M sequence failed to rescue VP40 budding above the basal level observed for the VP40 deletion mutant (data not shown). However, immunofluorescent confocal microscopy revealed that cells expressing VP40-NiV that contained the YPLGVG motif had a similar morphology to those expressing wild-type VP40, which was characterized by extensive filamentous projections that were frequently branching and fragmented (Fig. 3B). Martin-Serrano and co-workers, as well as others, previously observed this phenotype in cells expressing VP40 [41,48,49]. This cellular morphology was not observed when the native overlapping L-domains were deleted from VP40 (VP40 ΔPT/PY) (Fig. 3B). However, cells expressing wild-type NiV M showed a filamentous phenotype similar to those expressing VP40 (Fig. 4). In contrast, the NiV M Δ92–97 mutant, that was totally defective in budding, had an absence of the filamentous structures and appeared to localize at the nucleus. Cells expressing NiV M P93A displayed a somewhat intermediate phenotype with less pronounced filamentous structures and this phenotype also corresponded to the partial budding-defective phenotype (Fig. 4). Together, these results support the hypothesis that the NiV M-YPLGVG amino acid motif plays an important role in NiV M budding, and that it acts through a mechanism that is, in part, transferable to other viruses.

### NiV matrix budding is not dependent on VPS4A/B

**Figure 5:**
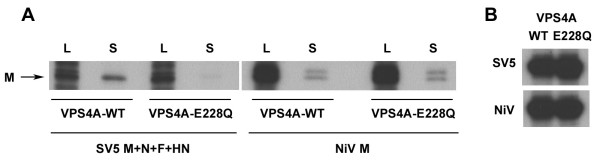
**NiV M release is insensitive to VPS4A inhibition**. (A) Cells were transfected with expression plasmids for SV5 N, M, F and HN, or NiV M, along with either wild-type or the dominant-negative VPS4A, followed by ^35^S-metabolic labeling. Released protein was pelleted through 10% sucrose, as described in the Methods, and proteins derived from cell lysates (L) and culture supernatants (S) were immunoprecipitated with either rabbit anti-SV5 polyclonal serum or MAb F45G5 (anti-NiV M) and analyzed by SDS-PAGE followed by autoradiography. (B) Expression of VPS4A was detected in cell lysates from the material analyzed in A by immunoprecipitation with rabbit polyclonal antiserum against GFP, followed by SDS-PAGE and autoradiography.

The involvement of certain components of the cellular MVB machinery has been demonstrated in several other viral systems (reviewed in [30-32]). A powerful technique that has helped facilitate the demonstration of these relationships has been the use of dominant-negative mutant versions of one or more of the protein components of the MVB complexes [31]. Dominant-negative (DN) mutants of the paralogous MVB proteins VPS4A and VPS4B have been shown to impair release of most viruses that use L-domains to accomplish budding [32]. To determine whether VPS4A has a functional role in NiV M VLP egress, we evaluated NiV M release in the presence of VPS4A-E228Q, a DN VPS4A mutant. SV5 VLP release, which is significantly reduced in the presence of DN VPS4A [38], was evaluated in parallel as a control. Cells were transfected with expression plasmids for SV5 proteins (M, N, F, and HN) or NiV M, along with 100 ng (per well) of plasmid containing Green Fluorescent Protein (GFP) fused to either wild-type or DN VPS4A. The quantities of the SV5 plasmids used were as described by Schmitt and co-workers [29] (N: 50 ng; M: 0.4 μg; F: 0.75 μg; HN: 0.75 μg), and 0.4 μg (per well) of NiV M was used along with empty vector for equivalent total DNA. Culture supernatants were clarified, and VLPs were centrifuged through a 10% sucrose cushion. Immunoprecipitation and SDS-PAGE analysis of the pellet revealed that SV5 VLPs (as indicated by M detection) were released in the presence of wild-type VPS4A, but not in the presence of DN VPS4A, as expected (Fig. 5A). In contrast, DN VPS4A had no effect on NiV M release (Fig. 5A). The difference observed between SV5 and NiV M release was not due to unequal VPS4A expression, which was equivalent in each group (Fig. 5B). NiV M release was similarly unaffected in the presence of DN VPS4B or both DN VPS4A and DN VPS4B (data not shown). These results suggest that NiV M VLP formation is not dependent on functional VPS4 proteins.

## Discussion

Previously, we observed that the M protein of NiV was capable of budding and forming VLPs when expressed independently in cell culture. In the present study we sought to characterize the NiV M protein further and explore whether NiV M possessed a classic L-domain motif that was required for efficient budding. Noting that all well-defined L-domains contain at least one proline residue, we initiated a mutagenesis strategy whereby individual proline residues in NiV M were mutated to alanine. We identified 3 sequences (4 proline residues, total) of interest because of their similarity to known L-domain motifs, or close alignment to the SV5 FPIV motif. Using this mutagenesis approach coupled with our NiV VLP budding assay, we identified one particular M mutant (P93A) that was defective in budding. To confirm this observation and further characterize the surrounding amino acids, we targeted the YPLGVG sequence within the M protein and additional alanine mutants were made (Y92A and L94A), as well as a deletion mutant (Δ92–97), which were then tested for budding ability. Whereas a low level of P93A release could sometimes be detected, we found the other YPLGVG mutants were completely defective in budding. This sequence motif is conserved in HeV and a P93A mutation in HeV M resulted in diminished budding ability.

To determine whether this sequence has transferable activity, the L-domain motifs and flanking residues of Ebola VP40 were replaced with the YPLGVG motif along with the appropriate NiV M flanking amino acids. Although budding of the VP40-NiV M recombinant containing the YPLGVG motif was not significantly restored, staining with anti-VP40 mAb and immunofluorescence microscopy revealed that cells expressing the VP40-NiV M recombinant containing YPLGVG displayed a morphology consistent with those expressing wild-type VP40 that was characterized by fragmented and branching filamentous structures. Similar morphology was observed for cells expressing wild-type NiV M, whereas mutation or deletion of the YPLGVG motif resulted in either less pronounced or an absence of the filamentous structures accompanied by nuclear localization of the mutant M protein. These data suggest that the YPLGVG sequence motif is important for proper NiV M cellular location and budding and can partially substitute for the native VP40 L-domain sequence. VPS4A and VPS4B are paralogous ATPases that are responsible for disassembly of the Endosomal Sorting Complex Required for Transport (ESCRT) complexes involved in MVB formation, and mutations that destroy their ability to bind or hydrolyze ATP result in the ability to dominantly inhibit cellular VPS4A or VPS4B. These DN mutants inhibited the budding and release of most viruses that use L-domains and are thought to provide a method of global inhibition of class E Vps proteins [30-32,50]. Notably, we found that NiV M budding and release was unchanged in the presence of DN VPS4A, while in parallel, SV5 VLP release was greatly reduced, in agreement with a previous report [38]. Furthermore, NiV M protein release was also unchanged in the presence of DN VPS4B, or in the presence of both DN VPS4A and DN VPS4B, thus eliminating the possibility that NiV M protein release was accomplished by VPS4B or that the wild-type cellular paralogs were rescuing release. We also observed that NiV M release is relatively insensitive to proteasome inhibition and unaffected by AIP1/Alix over-expression (data not shown), further suggesting that NiV M budding is independent of MVB components [31].

We took note of the YPLGVG sequence motif because of its similarity to the known L-domain YP(x)_n_L. This motif was first discovered in the Gag p9 protein of equine infectious anemia virus (EIAV) as YPDL [45] and then later identified in the p6 domain of human immunodeficiency virus type 1 (HIV-1) Gag protein (YPLTSL) [42]. Initial characterization of this motif in EIAV determined that the Y, P, and L residues were critical for virus particle budding; however, the motif was designated YxxL because of the similarity to the YxxL endocytosis motif, and it was hypothesized that the EIAV motif interacted with cellular endocytosis machinery [45]. However, it appears that AIP1/Alix is the primary protein that interacts with this L-domain, and the EIAV motif is now usually designated as YP(x)_n_L or YPxL because the proline residue is critical for AIP1/Alix binding and virus particle budding [30-32]. Both HIV-1 and EIAV Gag proteins contain a sequence just downstream of the YP(x)_n_L motif that is also important for AIP1/Alix binding, and it has been suggested that the two motifs are actually a single motif that can be summarized as (L) [F/Y]Px_1–3_LXX [I/L] [51].

Using the YxxL designation as a guide, Ciancanelli and Basler identified 62-YMYL-65 as a sequence important for NiV M budding [23]. Their study found that alanine mutation of Y62, Y62 and L65, or deletion of the whole sequence resulted in defective M protein budding, with deletion mutants exhibiting nuclear localization. They also established the functional rescue of L-domain-mutant VP40 budding by appending the YMYL sequence motif, with flanking elements to the C-terminus of VP40. We attempted to use this observation as a control for our experiments here by directly replacing the native VP40 L-domain with the identical NiV sequence used in that study, as we did with the present YPLGVG motif, rather than appending it to the C-terminus. However, this construct was also unable to restore budding of the L-domain-mutant VP40 (data not shown). The reason for the difference is not known; however, L-domains can be context-dependent, which may account for our differing result [31,40-42]. Interestingly, the equivalent sequence in HeV M is YMYM, and mutation of the NiV YMYL to the HeV sequence does not appear to adversely affect NiV M release (data not shown).

Based on their observation of nuclear localization of NiV M YMYL mutant, Ciancanelli and Basler speculated that NiV M contains competing trafficking signals, and that disruption of targeting to the plasma membrane resulted in the redirection of the protein to the nucleus [23]. Indeed, our observation of nuclear localization of the NiV M YPLGVG motif deletion mutant used here would be in agreement with this hypothesis, and we speculate that both sequences interact with one or more proteins in a common pathway, resulting in proper targeting of M. However, the mechanistic roles of either of these sequences in henipavirus budding remain to be clarified.

L-domains and their interactions with cellular MVB machinery, most likely represent a subset of protein domains involved in virus budding. An early observation made regarding Gag truncation mutants of HIV-1, which did not express the L-domain-containing p6 protein, was the apparent tethering of budding virions to the cell surface [31,52]. This phenotype appears to represent a defect in the final step of virus-cell separation and suggests that mechanisms other than those underlying L-domain function also play an important role in HIV-1 budding. In addition, deletion of the Ebola virus overlapping L-domains in VP40 results in a modest defect in replication competent virus production and suggests that mechanisms independent of MVB machinery may play a dominant role in Ebola virus budding [53]. The NiV YPLGVG sequence motif may facilitate budding of NiV M via interactions with non-MVB-associated cellular proteins, which enable the associated cellular morphology characterized by extensive filamentous projections that is also seen with VP40, by this alternative mechanism. The observation that NiV M release is insensitive to DN VPS4 proteins and proteasome inhibition lends some support to this interpretation. However, vesicular stomatitis virus (VSV) has been reported to be insensitive to DN VPS4A [44], and EIAV is insensitive to proteasome inhibitors [54-56]. Thus, insensitivity to DN VPS4 proteins and proteasome inhibitors may not be sufficient grounds to rule out L-domain activity. Further, it also remains a formal possibility that the NiV M protein YPLGVG sequence motif failed to rescue VP40 budding while restoring the filamentous cellular morphology because of the context of the flanking amino acids rather than a lack of intrinsic L-domain function.

Although studies with SeV have yielded conflicting results, Gosselin-Grenet and co-workers failed to find a reduction in SeV virion production in the presence of DN VPS4A or during suppression of AIP1/Alix expression. These results suggest that SeV budding also occurs independent of cellular MVB proteins, and are in agreement with the present observations on NiV M [47]. Chen and Lamb highlighted the reported VPS4 independence of VSV, SeV, and influenza virus and suggested that additional VPS4-independent viruses will serve as tools in uncovering the details of these additional mechanisms of virus budding [50], and our results suggest that NiV may also be a useful system to explore such alternative mechanisms. Further characterization of the YPLGVG sequence and whether there are any interactions with MVB cellular components in the context of both VLPs and live virus will be needed to definitively establish whether this element possesses classical L-domain activity or functions through some alternative mechanism.

## Conclusion

Using a recombinant expression system the budding process of the NiV M protein was examined and the amino acid motif YPLGVG within the M protein was found to be essential for M budding. Mutation or deletion of the YPLGVG motif also resulted in nuclear localization of NiV M. The transfer of the YPLGVG motif to an Ebola virus VP40 L-domain mutant did not restore its budding efficiency to wild type levels, but did restore the branched filamentous cell morphology characteristic of VP40 expressing cells. NiV M expression also resulted in the branched filamentous cell morphology, and YPLGVG motif NiV M mutant did not. Unlike classic L-domain containing proteins, we found no evidence of a role for MVB proteins in henipavirus budding. The data here suggest that whatever the specific role of the YPLGVG sequence has in NiV budding it appears distinct from the similar YP(x)_n_L sequence motif represented by EIAV. Further investigation of henipavirus assembly and budding may reveal a new mechanism of viral assembly and release that could be applicable to other enveloped viruses or have therapeutic implications.

## Materials and methods

### Cell lines

293T cells were maintained in Dulbecco's modified Eagle's medium (Quality Biologicals, Gaithersburg, MD) supplemented with 10% cosmic calf serum (Hyclone, Logan, UT), 2 mM L-glutamine, and 100 units/ml penicillin and streptomycin (Quality Biologicals, Gaithersburg, MD) (DMEM-10). COS-1 cells were maintained in Dulbecco's modified Eagle Medium Nutrient Mixture F-12 (Ham) 1× (Invitrogen, Carlsbad, CA) supplemented as described above (DMEM/F12-10).

### Antibodies

The following antibodies were used in immunoprecipitations: Monoclonal antibody (MAb) F45G5 (anti-M) [57] was kindly provided by Jody Berry and Hana Weingartl (National Centre for Foreign Animal Disease, Canadian Food Inspection Agency). Rabbit anti-SV5 serum was kindly provided by Robert Lamb (Northwestern University). Polyclonal sera from a rabbit immunized with gamma-irradiated NiV and a MAb directed against VP40 were also used. Rabbit polyclonal antisera against the green fluorescent protein (GFP) was purchased (Invitrogen, Carlsbad, CA).

### Plasmids

The creation of pCAGGS-NiV M has been described previously [22]. The HeV M ORF was PCR amplified from pCP436 (HeV M gene in pTD1) using the primers 5'-GTTTAAACCACCATGGATTTTAGTGTG (HEVMS) and 5'-GTTTAAACTCACCCCTTTAGGATCTTC (HEVMAS). PCR was done using Accupol DNA polymerase (PGS Scientifics Corp., Gaithersburg, MD) with the following settings: 94°C for 5 min, then 25 cycles of 94°C for 1 min, 55°C for 2 min, then 72°C for 3 min. The resulting PCR product was ligated into pCRII-Blunt-TOPO (Invitrogen, Carlsbad, CA), and then sub-cloned as a *Pme*I fragment into the pCAGGS/MCS *Sma*I site. Amino acid changes were introduced into NiV M and Ebola VP40 using standard PCR techniques or PCR site-directed mutagenesis using the QuickChange II Site-Directed Mutagenesis Kit (Stratagene, La Jolla, CA). pCAGGS-SV5 M, pCAGGS-SV5-NP, pCAGGS-SV5-F, and pCAGGS-SV5-HN were kindly provided by Robert Lamb. Wesley Sundquist (University of Utah) kindly provided pEGFP-VPS4A-WT, pEGFP-VPS4A-E228Q, pDsRed-VPS4B-WT, pDsRed-VPS4B-E235Q.

### Henipavirus matrix budding assay

NiV and HeV M VLP release was evaluated as previously described [22], with minor modifications. Briefly, 293T cells in 6-cm wells were transfected with 1 μg of each expression plasmid (unless otherwise stated) in duplicate using FuGene 6 transfection reagent (Roche, Indianapolis, IN) according to the manufacturer's instructions. At 24 h post transfection the culture medium was replaced with methionine-cysteine-free minimal essential medium (MEM) (Invitrogen, Carlsbad, CA) containing 2.5% dialyzed fetal calf serum (Invitrogen, Carlsbad, CA) and 100 μCi/ml ^35^S-cys/met Redivue Promix (Amersham Pharmacia Biotech, Piscataway, NJ). At 20–24 h p.t. the cell culture medium was removed, clarified, and then centrifuged through a cushion of 10% sucrose (w/vol) (unless stated otherwise) in NTE (100 mM NaCl; 10 mM Tris-HCl, pH 7.5; 1 mM EDTA) at 200,000 × g for 2 h at 4°C. Cells were removed by scraping and lysed in 200 μl lysis buffer (100 mM Tris-HCl, pH 8.0; 100 mM NaCl; 1.0% Triton-X 100) containing Complete, Mini protease inhibitors at a 1× concentration (Roche, Indianapolis, IN). Pelleted VLPs were resuspended in 200 μl lysis buffer, and proteins derived from lysates and supernatants were incubated with the appropriate antibody overnight at 4°C, followed by addition of Protein G-Sepharose (Amersham Biosciences, GE Healthcare, Piscataway, NJ). Proteins were analyzed by SDS-PAGE and autoradiography.

### Ebola VP40 budding assay

The expression plasmids for Ebola VP40 and glycoprotein GP have been generated as described previously [35,49]. A plasmid encoding the VP40-NiV protein chimera was produced by introducing the putative L-domain of NiV in place of the VP40 L-domain by PCR and inserted into vector pCAGGS using EcoRI and XhoI restriction endonucleases. All introduced mutations were confirmed by automated DNA sequencing. Human 293T cells in six-well plates were transfected with 2 μg plasmid DNA of Ebola GP plus 2 μg plasmid DNA of VP40WT or VP40-NiV chimera by using the Lipofectamine reagent (Invitrogen, Carlsbad, CA) and the protocol of the supplier. At 20–24 h post-transfection, proteins were metabolically labeled with 150 μCi of ^35^S Met-Cys (Perkin-Elmer, Wellesley, MA) for 5 h. Culture media was centrifuged at 2,500 rpm for 10 min to remove cellular debris, layered onto a 20% sucrose cushion in STE buffer (0.01 M Tris-HCl [pH 7.5], 0.01 M NaCl, 0.001 M EDTA [pH 8.0]), and centrifuged at 36,000 rpm for 2 h at 4°C. The resulting pellet containing VLPs was suspended in 100 μl of STE buffer followed by 300 μl of RIPA buffer (50 mM Tris [pH 8.0], 150 mM NaCl, 1.0% NP-40, 0.5% deoxycholate, 0.1% SDS) overnight at 4°C. The VLPs were immunoprecipitated with the anti-VP40 monoclonal antibody at 4°C overnight. The immune complexes were then precipitated with 50 μl of a 20% protein A agarose bead suspension and analyzed by SDS-PAGE. Protein bands were visualized by autoradiography and quantified by phosphorimager analysis.

### Indirect immunofluorescence

COS-1 cells were transfected with plasmids encoding VP40WT, VP40ΔPT/PY or VP40-NiV proteins using Lipofectamine and the protocol of supplier (Invitrogen, Carlsbad, CA), or with plasmids encoding WT NiV M, P93A, or Δ92–97 using FuGene 6 transfection reagent (Roche, Indianapolis, IN). The cells were fixed with 4.0% paraformaldehyde in 1× PBS (fresh-made) at room temperature for 10 min at 20–24 h post-transfection, washed three times with 1× PBS, permeabilized with 0.2% Triton X-100 in 1× PBS on ice for 10 min, and washed three times with 1× PBS. The cells were incubated with either mouse anti-VP40 MAb (1:100), or F45G5 (anti-NiV M) (3:100) in 3% BSA/PBS at 37°C in the dark for 30 min and washed three times, and then incubated with Alexa Fluor 488 donkey anti-mouse antibody (1:500) (Molecular Probes, Invitrogen, Carlsbad, CA) in 3% BSA/PBS at 37°C in the dark for 30 min. The cells were washed three times with 1× PBS and one time with water, and then mounted with Prolong antifade solution (Molecular Probes, Invitrogen, Carlsbad, CA) and analyzed by confocal immunofluorescent microscopy (VP40) or immunofluorescent microscopy (NiV M).

### Protein Sequence Alignment

ClustalW protein alignment was performed using the ClustalW program of the European Bioinformatics Institute  and the Jalview [58] multiple alignment editor was used to view the ClustalW alignment. Matrix gene sequences were derived from the following GenBank accession numbers: Nipah virus (NP_112025.1); Tupaia paramyxovirus (NP_054694.1); measles virus (NP_056921.1); Sendai virus (NP_056876.1); Newcastle disease virus (NP_071468.1); simian virus 5 (YP_138514.1). The Hendra virus matrix sequence was derived directly from pCP436.

## Competing interests

The authors declare that they have no competing interests.

## Authors' contributions

JRP conceived and contributed to the development of the NiV M budding assay, identified potential late domain-like elements in the M protein, designed and constructed all the M mutation-containing expression constructs and carried out the recombinant expression and analysis assays, interpreted data, and wrote the first draft of the manuscript. ZH and SEM designed and carried out the Ebola virus VP40 budding assays and interpreted the data. LY assisted in the design and execution of the immunofluorescence microscopy experiments. RNH provided supervision, financial support and edited and corrected the manuscript. LFW edited and corrected the manuscript. CCB conceived and contributed to the development of the NiV M budding assay and mutagenesis approach, provided overall supervision and financial support and wrote and prepared the final versions of the manuscript.
